# Women’s midlife health: the unfinished research agenda

**DOI:** 10.1186/s40695-023-00090-5

**Published:** 2023-10-03

**Authors:** Sioban D. Harlow, Lynnette Leidy Sievert, Andrea Z. LaCroix, Gita D. Mishra, Nancy Fugate Woods

**Affiliations:** 1 Department of Epidemiology, University of Michigan, 1415 Washington Heights, Ann Arbor, MI 48109-2029 USA; 2 Department of Anthropology, University of Massachusetts, Amherst, USA; 3 Herbert Wertheim School of Public Health and Human Longevity Science, University of California San Diego, San Diego, USA; 4 Australian Women and Girls’ Health Research Center, School of Public Health, University of Queensland, Seattle, Australia; 5 School of Nursing, University of Washington, Seattle, USA

**Keywords:** Women’s Health, Midlife, Aging, Middle Age, Menopause

## Introduction

In 2015 we launched Women’s Midlife Health with the aim of increasing scientific focus on the midlife -- a critical window for preventing chronic disease, optimizing health and functioning, and promoting healthy aging. This life stage, from about age 35–40 years to about 60–65 years, is coincident with the menopausal transition in women, encompassing the late reproductive to late postmenopausal stages of reproductive aging [1–4]. As noted in our initial editorial [5], in addition to the hallmark symptoms of menopause, the midlife is a vulnerable window for onset of gynecologic and hormone-sensitive conditions as well as onset or exacerbation of symptoms, symptom burden and functional limitations. It is also the critical window for interventions to reduce the loss of bone strength associated with menopause, to protect heart health and to promote psychosocial well-being. At the time we started this publication, relatively few journals were interested in manuscripts on midlife aging, except for a handful focused on menopause and reproductive aging. Women’s Midlife Health was designed to fill this gap. We developed an international community of investigators studying the intersection of menopause and midlife health who have contributed to enlarging scientific understanding, developing an international view of the perimenopause and acquainting investigators with studies of menopause around the world. Over the past decade, as scientific knowledge has increased, documenting the importance of the midlife for healthy aging, menopause journals have expanded their scope and aging journals have expanded their breadth. As multiple excellent venues are now available to publish work on midlife health, we have decided to cease publication of this journal. In our closing editorial, we provide reflections on what we have achieved and outline some of the continuing critical gaps in scientific knowledge.

## The unfinished research agenda

Scientific terminology for menopause and reproductive aging was initially defined only in 1980 [6]. At about the same time, social scientists were documenting unexpected variation in symptom experience within and across populations [7–9], alerting practitioners to a previously unrecognized need to understand the physiology and context of menopause and midlife. Concerted and well-funded research efforts to advance knowledge about the natural history of menopause, including data on its changing physiology and pathophysiology and their related biosocial changes, were initiated in the last decades of the twentieth century [10–14]. In the 1990’s and early 2000’s, the first wave of cohort studies elucidated the hormonal transitions that characterize ovarian aging and the specific symptoms attributable to them while documenting the inter-relationships between ovarian aging and bone, cardiovascular, and musculoskeletal aging. Over the last two decades these insights have led to the establishment of an evidence-based model for staging reproductive aging (i.e., the STRAW + 10 model ) [1–4], a developing theory of midlife aging with initial guidance as to critical windows for intervention, and an appreciation of variation in age at menopause and symptom experiences related to the complex interplay across physiologic and sociocultural systems that can influence, adversely or beneficially, the prospects for healthy aging. Moreover, foundational studies over the past three decades have laid the groundwork for understanding ovarian aging and its interface with other aspects of aging in midlife. Critical gaps in scientific knowledge remain that will require a new generation of well-funded studies. Some gaps represent a simple failure to ask basic questions, others signal a need for an expanded global focus encompassing further geographic and population diversity in the midlife experience, while others reflect the new research questions that have arisen from our current studies.

Although considerable information has been generated regarding the nature of ovarian aging for women who experience natural menopause, information for women experiencing surgical menopause (double oophorectomy), hysterectomy [15] and for women with chronic illnesses (e.g., HIV, cancer or autoimmune disease) that alter the menopause experience, remains lacking. Often excluded from study samples or analyses by design, yet likely to experience more abrupt hormonal changes and symptomatology, and more adverse health consequences, studies addressing the trajectories of ovarian aging and associated symptoms and pathophysiologic changes are warranted. Such studies are of particular importance given the differing risks of hysterectomy and surgical menopause by race, ethnicity and region of residence and the health risks associated with early menopause and premature ovarian insufficiency [16]. Also, given recent evidence of racial and ethnic differences in the timing of menopause, symptom experience and clustering of risk factors, considerably more focus on the midlife experience of minoritized populations and population specific risk factors, including the impact of structural racism, is of paramount importance [17].

Similarly, data on the midlife continues to remain limited from low- and middle-income countries, despite the important contribution of chronic diseases to the global burden of disease. As pointed out in our series on Contraception in Midlife, Demographic and Health Surveys, a major source of health information for many countries, only interview women of reproductive age, from ~ 15 to 49 years old [18]. Here, too, evaluation of context specific health risks common in such settings, such as the impact of high parity, early and late childbirth, increased risk of pelvic floor disorders, anemia, infectious diseases, high parasite load, life-long under-nutrition, and the increasing frequency of natural disasters associated with climate change is lacking. Other population subgroups for whom information on the menopausal transition experience and medical care options is limited are lesbian and transgender individuals.

As the STRAW + 10 model for staging reproductive aging [1–4] predates the generation of a substantive literature on the relationships between declines in anti-mullerian hormone (AMH), ovarian reserve and menopause, a revision of the STRAW + 10 model incorporating information on AMH levels across stages of the menopausal transition is overdue.

Also, given the substantive body of literature now published on the natural history of menopause and midlife health, knowledge translation to intervention trials and education of women and their health care providers is urgently needed. For example, the now well documented timing of precipitous bone loss during the late menopausal transition stages [19] argues for earlier screening of at-risk women and trials aimed at evaluating whether treatment across the trans-menopausal period (from approximately 2 years before until 1 year after the menopause) can prevent this precipitous loss. Similarly, most health care providers remain poorly informed about how to evaluate and treat bothersome menopausal symptoms, while much of what we have learned has yet to be made accessible to women themselves. This gap continues despite the publication of numerous rigorous randomized controlled trials that could easily be clinically translated so that women can choose, based on truthful comparisons and accurate information about the magnitude of benefits and risks, from many pharmacologic therapies that modestly improve vasomotor symptom frequency, severity, midlife sleep problems and menopause-related quality of life [20].

One intriguing insight gained over the last quarter century, is the complex interplay among symptoms and physiologic risk factors, suggesting that more attentions to bi- and multi-directional relationships among symptoms and heart, cognitive, and psychosocial health is warranted. For example, the relative timing of and feedback across endocrine changes, sleep disturbances, vasomotor symptoms and cognitive health remain poorly understood as does the inter-relationship between sleep, vasomotor symptoms, heart rate variability and cardiovascular risk.

We have learned a great deal about the types of life challenges facing midlife women from studies begun in the 1990s and later. Nonetheless, we need to remain vigilant to the unique and evolving stressors facing midlife women as they provide an important understanding of the social context for women’s health. Recent research about menopause and the workplace is just one dimension needing consideration, along with the pressures of family life (partners, children, and aging parents to name a few) [21–23].

Some further areas that have received limited attention to date and remain understudied include:

- The relationship between the increased menstrual flow experienced by most women during the menopausal transition -- often consistent with definitions of abnormal uterine bleeding -- and classic symptoms of menopause including fatigue, trouble focusing, depression and anxiety, heart palpitations and hot flashes;
- Risk factors for and pathophysiologic mechanisms underlying vasomotor symptoms that are not timed to changes in reproductive hormone levels, including those that begin long before and last long after the final menstrual period;
- Differences in the risks and consequences of hot flashes versus night sweats, two symptoms that are often combined into vasomotor symptoms but vary dramatically in experience and are identifiably different on ambulatory monitor graphs;
- The impact of exposure to endocrine disrupting chemicals, including forever chemicals, heavy metals and other environmental pollutants on endocrine function, musculoskeletal changes, heart health and cognition across the midlife;
- The relationship between menstrual disorders prior to midlife and symptoms experienced during menopause, and their impact, or not, on work productivity;
- Assessment of menopausal symptoms/outcomes across countries and cultures using both quantitative and qualitative surveys, collecting both symptom presence and symptom bothersomeness in the context of women’s lives;
- The role of structural racism, neighborhood conditions, and historical trauma on timing of menopause and risk of menopausal symptoms and on the symptom and health burden women carry as they enter the menopausal transition; and,
- Investigation of factors associated with positive menopausal transitions, including research on effects of education about menopause, provision of anticipatory guidance to help women manage the changes they are facing, and their development of attributions helping them sort the menopause-related to life-related factors.

As we approach the next generation of midlife studies, priority should also be given to advancing research methods, including in the following areas:

- As serial hormonal testing is not accessible to many women and in many countries, development of a validated bleeding questionnaire for staging reproductive aging;
- Building on core outcomes developed for vasomotor symptoms and genitourinary symptoms of menopause, definition of a core outcomes for additional symptoms of menopause and midlife health endpoints (https://medicine.unimelb.edu.au/school-structure/obstetrics-and-gynaecology/research/COMMA) [24, 25]; and,
- Given the complex interplay of multiple pathophysiologic changes that underlie symptoms and disease onset across the midlife, further development of statistical methods that permit simultaneous modeling of both the means and variances of multiple dynamic trajectories.

Finally, collaboration with health professional organizations is necessary to enrich their educational preparation to provide appropriate and helpful care to women in anticipation of and during the peri- menopause and post-menopause.

In closing, *Women’s Midlife Health* has met its goal of opening opportunities for publications on this critical lifestage and has published several important topical series on contraception, pain, stress, disability and structural racism in addition to innovative papers that have expanded the paradigm of and refocused the research agenda for women’s health. All papers will remain accessible at https://womensmidlifehealthjournal.biomedcentral.com/ and on PubMed https://pubmed.ncbi.nlm.nih.gov/. We thank all the authors and reviewers who contributed to the success of the journal.
